# Unusual Case of Acute Coronary Syndrome Due to Compression of the Left Main Coronary Artery From a Contained Aortic Root Perforation

**DOI:** 10.7759/cureus.43492

**Published:** 2023-08-14

**Authors:** Victor H Molina-Lopez, Porfirio E Diaz-Rodriguez, Juan J Rivera-Torres, Juan Vazquez-Fuster, Joseph Maldonado-Suarez, Sonia Vicenty-Rivera

**Affiliations:** 1 Cardiology, Veterans Affairs Medical Center, San Juan, PRI; 2 Cardiology, Percutaneous Coronary Intervention (PCI) Cardiology Group, San Juan, PRI

**Keywords:** extrinsic coronary artery compression, acute coronary syndrome, aortic root perforation, left sinus of valsalva pseudoaneurysm, aortic root pseudoaneurysm, left main coronary artery stenosis

## Abstract

We present an intriguing and rare case of a 71-year-old male who presented with a non-ST elevation myocardial infarction (NSTEMI). Initial coronary angiography revealed severe and unusual systolic extrinsic compression of the left main coronary artery (LM), warranting further advanced imaging investigations. Computed tomography angiography (CTA) and transesophageal echocardiography (TEE) were employed to determine the underlying cause, which was identified as a contained aortic rupture leading to the formation of a pseudoaneurysm in the left coronary sinus of Valsalva and aortic root. This condition was found to be a sequela of previously undiagnosed endocarditis, likely secondary to lower extremity osteomyelitis and bacteremia, for which the patient received prolonged intravenous (IV) antibiotic therapy. This case highlights the critical role advanced imaging techniques play in accurately diagnosing and characterizing complex cardiovascular abnormalities, enabling early intervention and optimizing patient outcomes. Healthcare providers should remain vigilant for such atypical presentations to ensure timely and appropriate management.

## Introduction

Myocardial ischemia and infarction resulting from extrinsic compression of the left coronary artery are exceedingly rare and have only been documented in a limited number of case reports [1-6]. This condition has been observed in patients with severe pulmonary artery dilation associated with pulmonary hypertension, anomalous origin of the left coronary artery from the right aortic sinus with an interarterial course, congenital aortic sinus of Valsalva aneurysms, pseudoaneurysms following aortic or cardiac surgery, and as acute or delayed complications of endocarditis [4,5]. Notably, aortic valve endocarditis can give rise to various subaortic and periaortic structural complications. In this case report, we present the unique instance of a patient with undiagnosed prosthetic aortic valve endocarditis who presented with acute coronary syndrome due to extrinsic compression of the left main (LM) coronary artery, resulting from a contained perforation and pseudoaneurysm of the left coronary sinus of Valsalva and aortic root. 

## Case presentation

**Figure 1:**
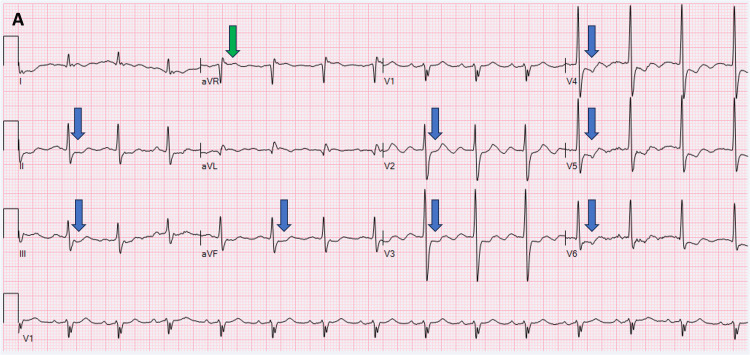
Twelve-lead ECG demonstrating ST elevation in aVR (green arrow) with ST depression (blue arrows) on inferior and anterolateral leads in the setting of acute coronary syndrome suggestive of ischemia due to the left main coronary artery (LM). ECG: electrocardiogram.

A 71-year-old male with a complex medical history presented to the emergency department complaining of multiple intermittent episodes of severe retrosternal and oppressive chest pain, accompanied by shortness of breath. The patient's past medical history included aortic valve replacement with a mechanical St. Jude #23 prosthesis (Abbott Laboratories, Chicago, USA) two years prior with coronary artery bypass grafts. Grafts were attached from the left internal mammary artery (LIMA) to the left anterior descending coronary artery (LAD), along with a saphenous vein graft (SVG) to the right coronary artery (RCA) and diagonal 1 (D1). He also had atrial fibrillation, arterial hypertension, type 2 diabetes mellitus, chronic kidney disease stage 4, and chronic warfarin use. The patient had a history of prolonged hospitalization and was completing intravenous (IV) antibiotics at a skilled nursing facility for osteomyelitis with bacteremia caused by vancomycin-resistant *Enterococcus faecium*. A transthoracic echocardiogram (TTE) performed during that admission failed to show any valvular vegetation, valvular structural damage, or valvular malfunction. He was on a regimen of multiple medications, including amiodarone, metoprolol tartrate, isosorbide mononitrate, warfarin, aspirin, atorvastatin, hydralazine, torsemide, and amlodipine. The patient completed 12 weeks of tailored antibiotic therapy. The patient's electrocardiogram (ECG) displayed ST depressions in the inferior and anterolateral leads, accompanied by elevated aVR (Figure 1).

**Figure 2:**
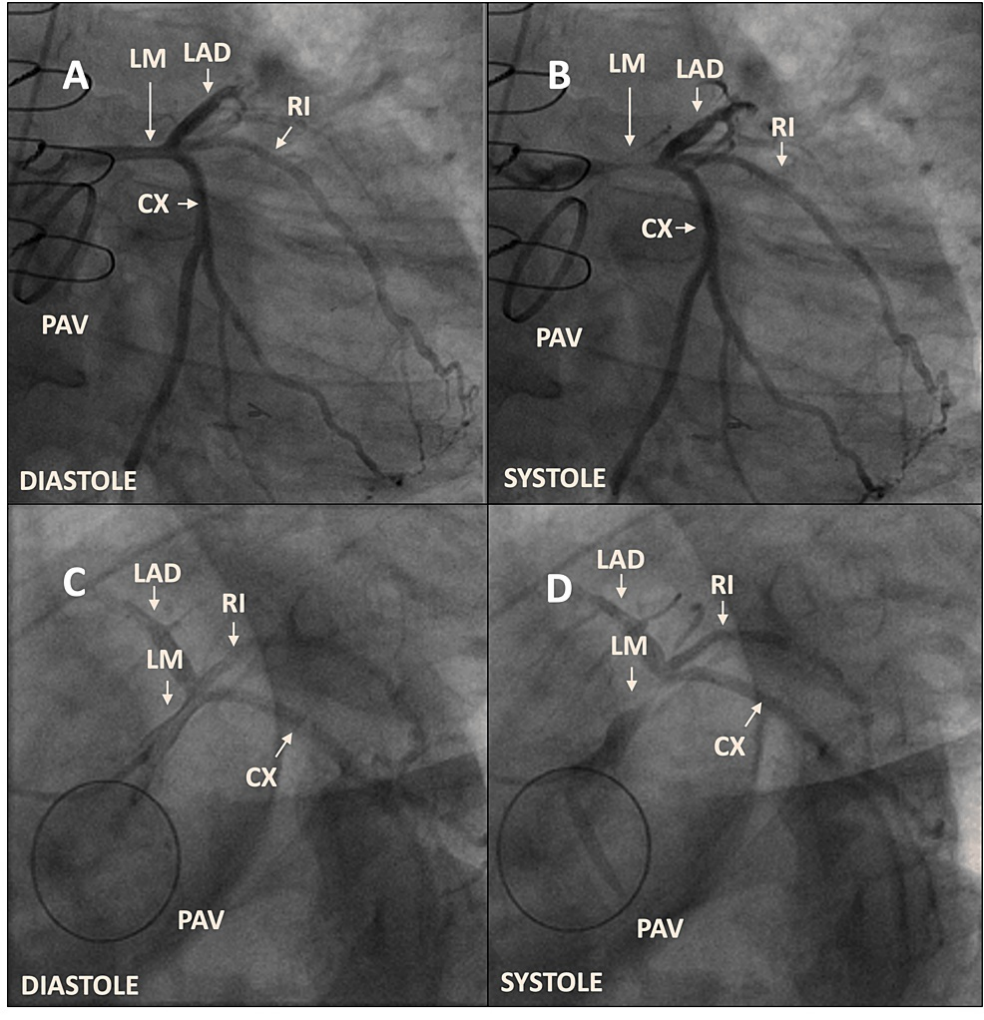
Right anterior oblique (RAO) 20° caudal 20° and left anterior oblique (LAO) 30° caudal 40° revealing mild stenosis of the left main coronary artery (LM) in diastole (A) and (C) with severe circumferential LM stenosis in systole (B) and (D). LAD: left anterior descending coronary artery.

High-sensitivity troponins were consistent with acute coronary syndrome (ACS). Due to the presence of acute chest pain consistent with angina, elevated cardiac markers, and ECG changes suggestive of ischemia, the patient underwent urgent coronary and graft angiography. Angiography revealed that the grafts were patent, but it also exposed severe systolic compression of the mid- to distal sections of the LM coronary artery (Figures 2A-2D; Video 1). Notably, this compression was not evident in the preoperative coronary angiogram, raising suspicion of a potential extrinsic compression mechanism.

**Video 1:** Left anterior oblique (LAO) with caudal angulation with systolic left main (LM) coronary artery compression.

**Figure 3:**
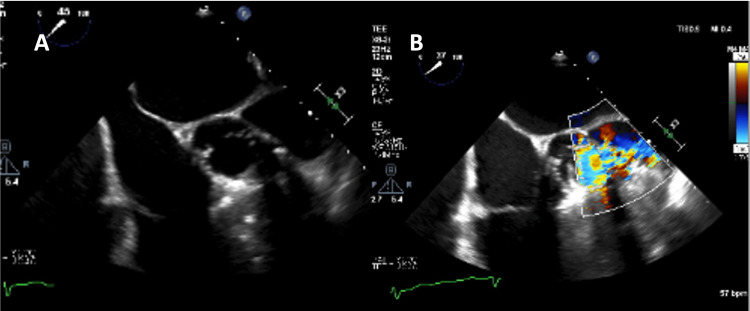
Transesophageal echocardiography demonstrating left coronary sinus of Valsalva and aortic ring rupture with pseudoaneurysm (A) and systolic flow by color flow Doppler (B).

In light of the patient's renal disease, a transesophageal echocardiogram (TEE) was done initially, which suggested the presence of a pseudoaneurysm in the aortic root with two entry points from the left coronary sinus of Valsalva (Figures 3A, 3B; Video 2). 

**Video 2:** Transesophageal echocardiography mid-esophageal short-axis view with color flow Doppler demonstrating the left coronary sinus of Valsalva contained perforation with systolic flow into the pseudoaneurysm.

**Figure 4:**
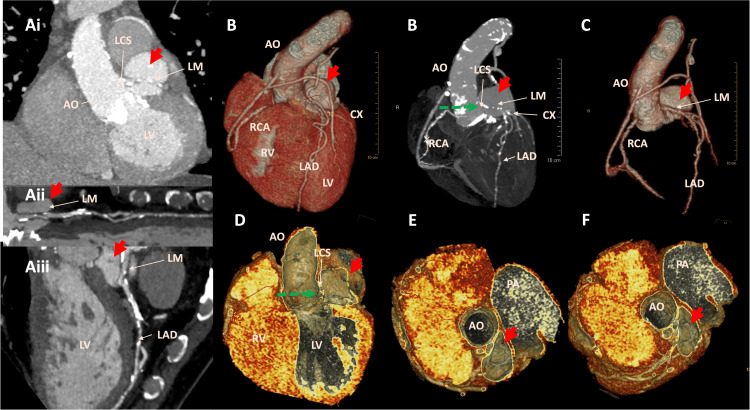
Gated cardiac computerized tomographic angiography (cCTA) with multiplanar reconstruction demonstrating the contained aortic root rupture and left coronary sinus of Valsalva pseudoaneurysm (red arrow) causing extrinsic compression of the LM coronary artery during systole. LM: left main coronary artery.

Further evaluation with gated cardiac computerized tomographic angiography (cCTA) revealed an extraluminal contrast-filled cavity adjacent to the left-sided aortic root, measuring 2.4 cm × 3.8 cm × 3.6 cm, and multiple peri-, supra-, and infra-valvular perforations (Figures 4A-4F). 

Based on the imaging findings, the patient was suspected to have a contained rupture of the aortic root, which encased the LM coronary artery and resulted in its collapse during systole, leading to angina and myocardial ischemia. The case was thoroughly discussed by the heart team and evaluated by cardiothoracic surgeons to determine the most appropriate course of action. After careful consideration, the patient underwent proximal aortic root repair along with homograft aortic valve repair. This surgical intervention aimed to address the aortic root rupture and restore normal blood flow to the compressed left main coronary artery. Post-operative management involved close monitoring in the intensive care unit, with a focus on optimizing cardiac function and preventing complications. Additionally, the patient's complex medical history required a comprehensive care plan involving collaboration among various specialties to ensure a successful recovery.

## Discussion

Aneurysms of the sinus of Valsalva can be classified as either congenital anomalies or acquired conditions. Congenital sinus of Valsalva aneurysms are rare and occur due to the absence of muscular and elastic tissue in the aortic wall of the sinus of Valsalva [4,5]. Conversely, the acquired sinus of Valsalva aneurysms can develop from various factors, including infections like bacterial endocarditis [1-3], degenerative diseases such as atherosclerosis and connective tissue disorders, and traumatic events [4-6]. Pseudoaneurysms can arise when the intimal layer of the sinus ruptures, leading to blood accumulation within a false lumen covered by the outer adventitia. These pseudoaneurysms are more likely to form due to localized intimal disease, trauma during medical procedures or surgeries, or infectious endocarditis or perivalvular abscess [1-6].

Our case presents an exceptional occurrence of non-ST elevation myocardial infarction (NSTEMI) caused by extrinsic compression of the LM coronary artery due to a contained aortic root perforation and a left coronary sinus pseudoaneurysm. This exceedingly rare condition emerged as a late consequence of previously undiagnosed prosthetic aortic valve endocarditis. Notably, there have been no reported cases of such extrinsic compression of the LM coronary artery to date. The unique etiology highlighted in this report underscores the significance of considering rare conditions in patients presenting with acute coronary syndromes, necessitating tailored management approaches for uncommon scenarios. Ultimately, this case report emphasizes the value of interdisciplinary collaboration and careful evaluation of atypical presentations to enhance patient care in complex cardiovascular cases. By sharing and analyzing such cases, we can deepen our understanding of rare cardiovascular complications and improve patient outcomes.

## Conclusions

This case highlights the vital role of advanced imaging techniques in accurately diagnosing and characterizing complex cardiovascular abnormalities, enabling early intervention for better patient outcomes. The unique etiology discussed emphasizes the importance of considering rare conditions in patients presenting with acute coronary syndromes, requiring tailored management approaches for such uncommon scenarios. Overall, this case report underscores the value of interdisciplinary collaboration and careful evaluation of atypical presentations to enhance patient care in complex cardiovascular cases.
